# An All-Dielectric Metamaterial Terahertz Biosensor for Cytokine Detection

**DOI:** 10.3390/mi15010053

**Published:** 2023-12-26

**Authors:** Kuo Men, Ziwei Lian, Hailing Tu, Hongbin Zhao, Qianhui Wei, Qingxi Jin, Changhui Mao, Feng Wei

**Affiliations:** 1 State Key Laboratory of Advanced Materials for Smart Sensing, GRINM Group Co., Ltd., Beijing 100088, China; menkuo@grinm.com (K.M.); jinqingxi@foxmail.com (Q.J.); mao@grinm.com (C.M.);; 2 GRIMAT Engineering Institute Co., Ltd., Beijing 101402, China; 3 GRINM (Guangdong) Institute for Advanced Materials and Technology, Foshan 528051, China; 4 General Research Institute for Nonferrous Metals, Beijing 100088, China

**Keywords:** all-dielectric metamaterial, terahertz biosensor, cytokine

## Abstract

In this paper, we report an all-dielectric metamaterial terahertz biosensor, which exhibits a high Q factor of 35 at an 0.82 resonance peak. A structure with an electromagnetically induced transparency effect was designed and fabricated to perform a Mie resonance for the terahertz response. The biosensor exhibits a limit of detection of 100 pg/mL for cytokine interleukin 2 (IL-2) and a linear response for the logarithm of the concentration of IL-2 in the range of 100 pg/mL to 1 μg/mL. This study implicates an important potential for the detection of cytokines in serum and has potential application in the clinical detection of cytokine release syndrome.

## 1. Introduction

Cytokine release syndrome (CRS) is one of the important causes of death in critically ill patients [1,2], which refers to an extreme immune response due to an over-activated or out-of-control immune system that releases a large amount of cytokines when a virus invades the human body.

Cytokines are a class of small-molecule soluble peptide proteins secreted by immune cells. Clinical studies have found that COVID-19 intensive care patients exhibit significantly elevated serum pro-inflammatory cytokine levels; interleukin 2 (IL-2) is the one of the typical cytokines [3,4].

Detecting CRS-related cytokines in patients’ serum samples and intervening early in the inflammatory response before the occurrence of severe CRS is an important part of clinical diagnosis, which is an important guide to correctly predetermine therapeutic guidance. Due to the low concentration of cytokines in serum (pM range), high-sensitivity biosensors are required for their detection.

The terahertz (THz) metamaterial biosensor is a non-destructive, label-free, highly sensitive sensor for pM-scale cytokine detection. However, most typical metamaterials are metal-based array structures, and the low Q factor of the device limits the sensitivity of the sensor due to high metal losses. Compared to metal-structured metamaterials, dielectric metamaterials have much lower losses, higher Q factors, and can be used as THz metamaterial biosensors to dramatically increase the sensitivity of the sensors and limit of detection. The Fano resonance is taken into account to further improve the device’s Q-factor, for example, dielectric Fano resonant structures based on silicon nanostripe [5], asymmetric-cut wire metamaterials [6], and an all-dielectric bound state in the continuum [7]. Yang creatively reported a silicon-based double-gap split-ring structure metamaterial in the near-infrared regime with a high Q factor of 483 which relied on a fano-type interference [8]. However, none of these high-Q-factor metamaterials have been applied to the THz band.

In this work, we designed and fabricated a dielectric metamaterial biosensor using a bonding process, so the dielectric layer is monocrystalline silicon and it avoids the electron beam lithography (EBL) exposure process, which enhances device reliability, reduces preparation costs and facilitates mass production. The limit of detection (LOD) of our device is about 100 pg/mL, which is lower than that of the gold nanobump arrays (LOD is about 20 ng/mL) [9] and the elliptic silicon disk-pair periodic arrays (LOD is about 0.002 g/L) [10] and is comparable to that of metal split-ring resonators (LOD is about 0.1 ng/mL) [11]. This study provides a feasible option for clinical CRS detection.

## 2. Material and Methods

### 2.1. Design and Fabrication of the THz Biosensor

In order to get large Q factor device, the dielectric layer and substrate must have a large difference in dielectric constant. The dielectric constant of silicon is about 11.7 and BF33 is about 4.6 in the range of 0.5 THz to 1 THz, which is a relatively large difference. This property can limit the propagation of electromagnetic waves, so that it is confined to the silicon structures. On the other hand, the BF33 glass has a relatively good transmittance in the THz band. Furthermore, the bonding of silicon wafer and BF33 glass is a frequently used wafer-level bonding technology; the process is mature, the equipment is inexpensive, and it is easy to be mass-produced. These advantages result in high device stability and consistency. Due to the presence of various impurities and defects in the BF33 glass, the thickness is too large to affect the transmittance of the material in the THz band, thus affecting the device performance, so it is necessary to select the appropriate thickness of the BF33 substrate. For the general electromagnetic wave propagation in the medium occurring in the Mie resonance, the three dimensions of the single resonant units are similar and their size is about 1/10 to 1/2 of the wavelength. So, the thickness of the silicon layer of the device ranges from dozens of microns to hundreds of microns thick. In addition, due to the bonding process, the upper layer of silicon wafers are too thin for the device preparation success rate and the yield is not high; on the other hand, the procurement of raw materials needs to be considered in the process of the economy of the problem. Therefore, comprehensive theoretical and practical operation of the two aspects is necessary: the optimal thickness of the silicon wafers is set to 200 μm, and the optimal thickness of the BF33 glass is set to 300 μm. Since the thickness of the etched silicon layer is 200 μm, the minimum transverse width is 8 μm, and the depth-to-width ratio reaches 25:1; in order to realize this depth-to-width ratio, the most classic of the deep-silicon etching processes, the Bosch process, should be used.

**Figure 1:**
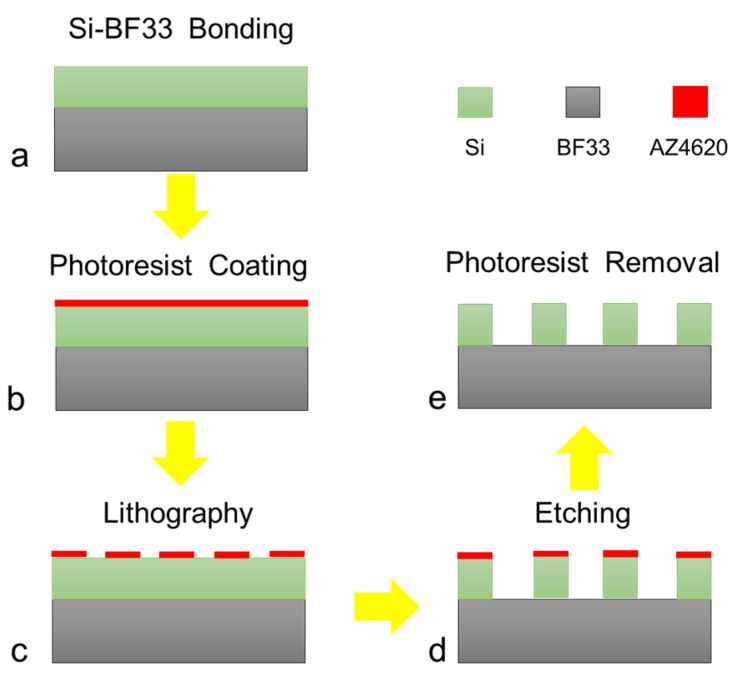
Fabrication process of the biosensor. (**a**) Bonding the silicon wafer and BF33 glass, (**b**) coating the photoresist AZ4620, (**c**) lithography and developing, (**d**) etching the double-gap ring column array, (**e**) removing the photoresist.

The fabrication flowchart of the biosensor is shown in Figure 1. The Si(100) wafer and BF33 glass (Schott Borofloat33) both with a thickness of 200 μm were used for anodic bonding, in which the silicon wafer was double polished with a resistivity of 10,000 Ω·cm. The AZ4620 photoresist was spin coated and patterned using standard photolithography for about 4 µm. Then, the photoresist was hard baked at 110 °C for 20 min for the next dry etching step. 

A dry etching process was utilized to fabricate the double-gap ring column arrays with a height of 200 µm by the Bosch process. The flow rate of C_4_F_8_ and SF_6_ in a single cycle is 4:5, etching for 400 cycles. Finally, the photoresist was removed and cleaned, and the surface of the device was obtained. 

### 2.2. Surface Modification

**Figure 2:**
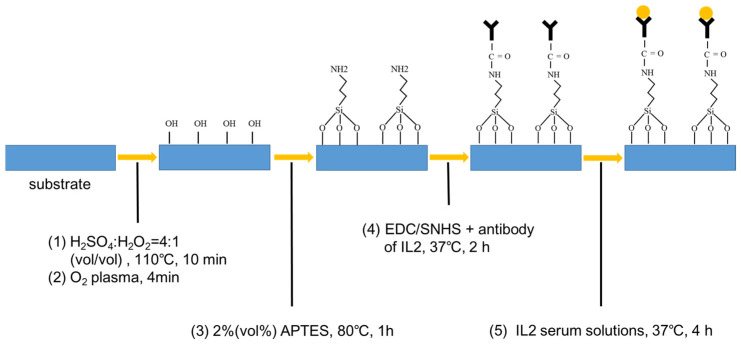
The schematic of surface functionalization of the device.

To improve the sensitivity and specific detection of IL-2 (Sangon Biotech, Shanghai, China), the surface of the biosensors should be functionalized by surface modification [12], as depicted in Figure 2. 

Before surface modification, the device was cleaned by a solution mixture of H_2_SO_4_: H_2_O_2_ in a volume ratio of 4:1 at 110 °C for 10 min to ensure the reliability of the functionalized modifications on the surface of silicon. 

First, oxygen plasma treatment (ICP plasma, 4 min) was performed to produce more terminal hydroxyl groups (-OH) on the surface of silicon. Then 2% (*v*/*v*) 3-aminopropyltriethoxysilane (APTES, Sigma-Aldrich, St. Louis, MO, USA) was soluted in absolute ethanol as a crosslinker solution to form amino groups (-NH_2_) on the surface of silicon. After immersion in APTES solution for 30 min, the biosensors were rinsed with ethanol three times and annealed at 120 °C for 30 min to achieve a firm silanization. Then, carbodiimide (EDC, Sigma-Aldrich) and N-hydroxysulfosuccinimide (sNHS, Sigma-Aldrich) were dissolved in fetal bovine serum (Sangon Biotech, E51002) at a final concentration of 10 mg/mL which was used as a coupling agent. Afterwards, the devices with -NH_2_-functionalized surfaces were dipped into the coupling agent solution for 1 h at room temperature. In the following step, the biosensors were dried under a N_2_ flow to remove the residual water. 

After the surface functionalization procedure, the IL-2 antibody (Sangon Biotech) was added onto the fabricated biosensors and incubated at 37 °C for 1 h under humid conditions. Herein, IL-2 serum solutions were coated on the surfaces of the devices modified with the corresponding antibodies, respectively. Given the strong absorption of terahertz waves by water molecules, the biosensors needed to be dried out after dropping the IL-2 solution.

### 2.3. Measurement and Simulation Method

The microstructure of the device was characterized using a scanning electron microscope (SEM). The modification of the device surface was tested using the atomic force microscope (AFM) and X-ray photoelectron spectroscopy (XPS). The biosensor measurements were performed on a commercial Fourier infrared spectrometer system (Brucker) to get the THz transmission spectra. Due to the strong absorption of THz waves by water molecules, all of our measurements were carried out in a dry environment. The tests were completed within two hours to ensure the biological activity of the cytokines. The frequency shifts (∆f) could be obtained easily by monitoring the relative changes in the resonance peaks of transmission spectra between different samples. Each sample was measured three times repeatedly. Full-wave numerical simulation was performed using the commercial software comsol. 

## 3. Results and Discussion 

### 3.1. The Microstructure of the Device and the Certification of Modification 

**Figure 3:**
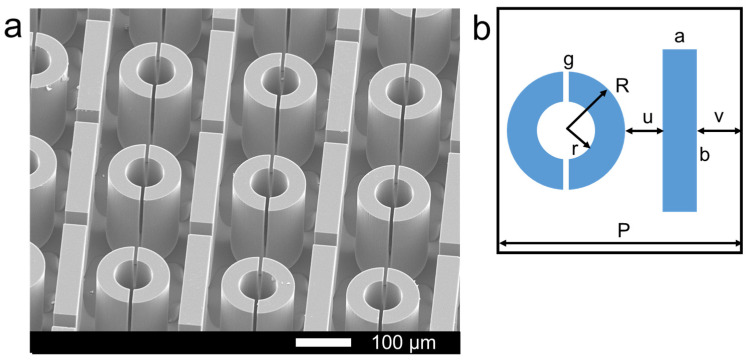
(**a**) The SEM image of the biosensor, (**b**) the size of the unit cell.

The microstructure of the device is shown in Figure 3. The SEM image of the biosensor is shown in Figure 3a, and the size of the unit cell is shown in Figure 3b, in which a = 50 μm, b = 240 μm, g = 8 μm, R = 75 μm, r = 37 μm, u = 24 μm, v = 27 μm, P = 250 μm. 

**Figure 4:**
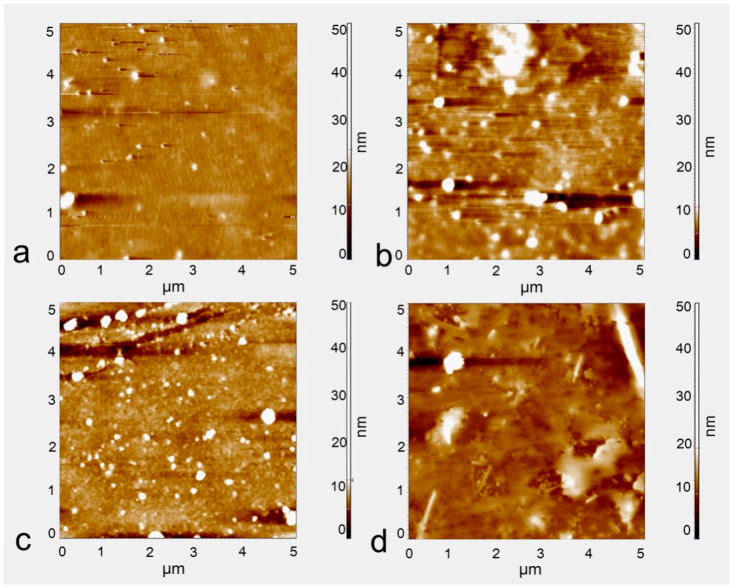
AFM image at different functionalization steps. (**a**) The surface of the substrate, (**b**) after oxygen plasma treatment, (**c**) after reaction with APTES and coupling agent solution, (**d**) modification of IL-2 antibody.

The immobilization of IL-2 antibodies on the silicon surface was achieved by the method described above. The modification processes were examined by AFM measurements. AFM images of the biosensor surface at different functionalization steps are shown in Figure 4. The pristine double-gap ring column array exhibits relatively flat surfaces (Figure 4a). Then, with the oxygen plasma treatment, the surface of the silicon enriched more terminal hydroxyl groups (-OH) (Figure 4b). After incubation with a solution of APTES for 30 min, the immobilization of silanes onto the silicon surface can be clearly observed (Figure 4c), causing the surface roughness value to increase. After the immobilization of the IL-2 antibody, the surface of the biosensor is coated with large particles, suggesting that the antibody is indeed attached by the pre-adsorbed -CHO groups (Figure 4d). The average surface roughness values of the four samples are 0.827 nm, 1.355 nm, 1.989 nm, 10.320 nm, respectively. The increase in roughness reflects that the device surface has been successfully modified by the antibody to some degree.

**Figure 5:**
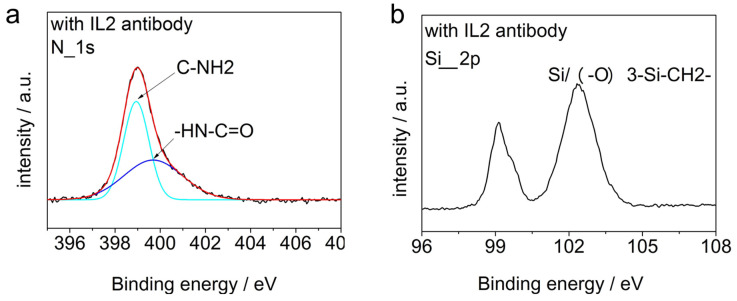
*XPS* spectra analysis for the modified device. (**a**) The N_1s_ peak of the sample modified by the IL-2 antibody and the results of its split-peak fitting, (**b**) the Si_2p_ peak of the sample modified by the IL-2 antibody.

In order to confirm that the silicon surface was decorated by IL-2 antibodies further, *XPS* spectra analysis was carried out on the samples modified by the antibodies, which is shown in Figure 5.

In the XPS images, the peptide bonds (-HN-C=O) formed by dehydration condensation are observed in Figure 5a and siloxane bonds (Si/(-O)_3_-Si-CH_2_-) are observed in Figure 5b. This demonstrated that the device surfaces were modified by the antibodies successfully.

### 3.2. The Simulation and Experimental Results of the Device 

**Figure 6:**
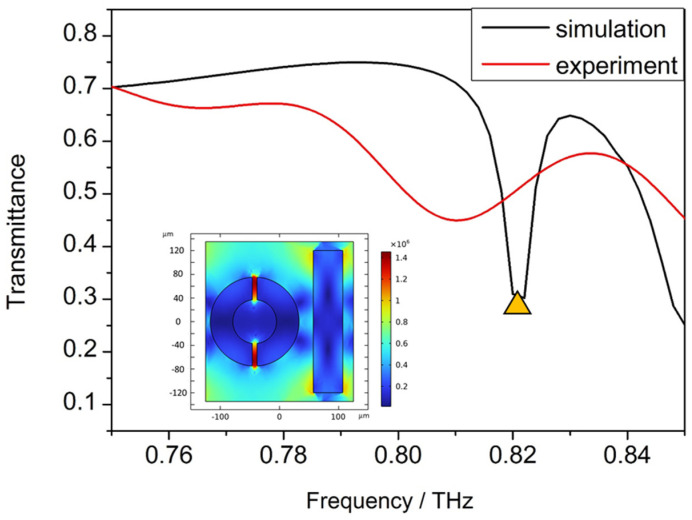
Simulated and experimental transmission spectrum of the device, the distributions of the electric fields at 0.82 THz marked by yellow triangle shown in the insert.

Numerical simulations have been performed to analyze the peaks of the transmission spectrum, and the distribution of the electric field to clarify the resonant modes was obtained clearly. The simulated transmission spectrum is shown in Figure 6 and the distributions of the electric fields perpendicular to the axial at a 0.82 THz resonance peak are shown in the inserts. A significant electric field enhancement occurs at both gaps of the ring.

The resonance peak positions of the simulation results and the experimental results are the almost the same, but the Q factor of the device in the simulation results is obviously larger than that in the experimental results, which is hard to avoid. The reason for limiting the actual Q factor of the device may be due to imprecise processing, or it might be due to the minimum sampling step of the Fourier infrared spectrometer system of 0.0003 THz. 

The advantage of all-dielectric metamaterials over metal-based metamaterials is the low dielectric loss. When the electromagnetic wave with a frequency below or near the bandgap frequency of the material hits a high-index dielectric sphere or cylinder, both the magnetic and electric dipole resonances are excited, making the particles behave like a magnetic dipole (first Mie resonance) and an electric dipole (second Mie resonance) [13]. This mechanism of the resonance avoids electromagnetic loss effectively and achieves higher Q factors since the displacement currents in dielectric resonators do not exhibit thermal effects [14].

On this basis, we have designed an all-dielectric structure that resembles an electromagnetically induced transparent (EIT) structure. The structure is formed from a periodic lattice made of a rectangular bar resonator and a ring resonator, both formed from silicon. The rectangular bar resonator serves as an electric dipole antenna which couples strongly to free space excitation with the incident E-field oriented along the x-axis. The collective oscillations of the bar resonators form the “bright” mode resonance. The magnetic dipole mode in the ring cannot be directly excited by light at normal incidence as the magnetic arm of the incident wave is perpendicular to the dipole axis. However, it can couple to the bright mode bar resonator. Furthermore, the ring resonators interact through near-field coupling, resulting in collective oscillation of the resonators and suppression of radiative loss, forming the “dark” mode of the system. The interference between the collective bright and dark modes forms a typical 3-level Fano-resonant system [15], thus forming a resonance enhancement [16].

Accompanying the narrow transmittance curve, the metasurface also possesses a large electric field enhancement within the split-ring resonators, as is shown in the insert of Figure 6. The simulated field enhancement is on the same order as state-of-the-art nonlinear plasmonic devices. The major benefit of the dielectric metasurface, when comparing with its plasmonic counterparts, is that the field enhancement occurs within the volume of the dark mode resonator with excellent modal overlap with the silicon. The metasurface possesses the advantages of waveguide-based devices such as a large Q factor and good modal overlap with the active material while greatly reducing parasitic losses.

### 3.3. The Transmission Spectra and Sensitivity Analysis

**Figure 7:**
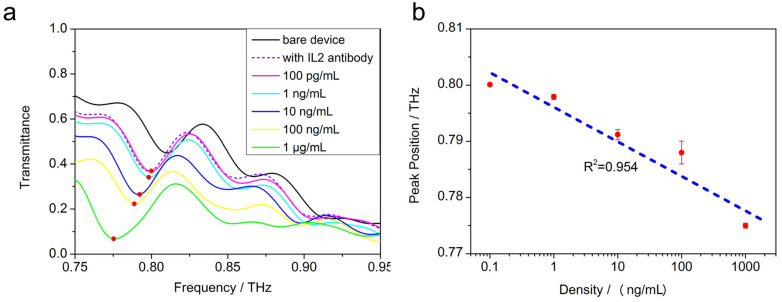
(**a**) The transmission spectra of the sensor device for different concentrations (100 pg/mL to 1 μg/mL) of IL-2 in serum. (**b**) The variation in frequency shift for two peaks with different concentrations of IL-2 and the sensitivity fitting curve.

To demonstrate the ability of the novel sensor device to detect lower-molecular-weight biomolecules, different concentration gradients of IL-2 serum solutions were prepared. The samples were dried naturally after they had been dropped on the devices. Then, the device was rapidly examined using the Fourier infrared spectrometer to get the transmission spectra, which are shown in Figure 7.

From Figure 7, the Q factors of the sensor can be extracted. The Q factor is commonly used to describe the narrow resonance features and can be estimated by $Q=f_{0}/\Delta f,$ where Δf is the fullwidth at half maximum (FWHM) of the resonance intensity and $f_{0}$ is the resonance frequency. Thus, the Q factors are about 35 at 0.82 THz.

As is known, sensitivity is a key parameter of sensors, which is defined as S = ∆f/∆n, where ∆f is the frequency shift, and ∆n is the refractive index change. The refractive index has a linear relationship with concentrations in the concentrated solution while it is nonlinear in the extreme diluted solution [17]. Hence, Sreekanth proposed the average equilibrium population N(c) of adsorbed particles on the sensor surface; in order to estimate the sensitivity conveniently, they determined a reliable upper bound on N(c), called N(c)_max_. They also pointed out that the relationship between N(c)_max_ and Δλ could be described by a two-exponential function phenomenologically [15]. Sreekanth’s idea was to fit the device sensitivity to the full spectrum, so a double exponential function was considered, but according to the fitted curves in our experiment, the sensitivity curve can be viewed as linear at low concentration conditions.

As the concentration increases, the resonance peak shifts to the left, and when the concentration of the IL-2 sample is as low as 100 pg/mL, the THz transmittance curve almost coincides with the device without the test samples, which proves that the biosensor reaches the limit of detection.

We found that the frequency shift ∆f has an approximate linear relationship with the logarithm of the concentration in the range of 100 pg/mL to 1 μg/mL. For the detection of IL-2, the resonant frequency peak position f and the concentration c satisfies the following equation (Equation (1)):

f = −0.0064 × lg(c) + 0.80744(1)

The slope of the fitted equation represents the relationship between the resonance peak frequency and the concentration of the biosamples, which can be used to characterize the sensitivity of this biosensor. In this way, complex nonlinear fitting was not required for the sensitivity calibration. The linear R^2^ is about 0.954.

### 3.4. Specificity of THz Biosensors

**Figure 8:**
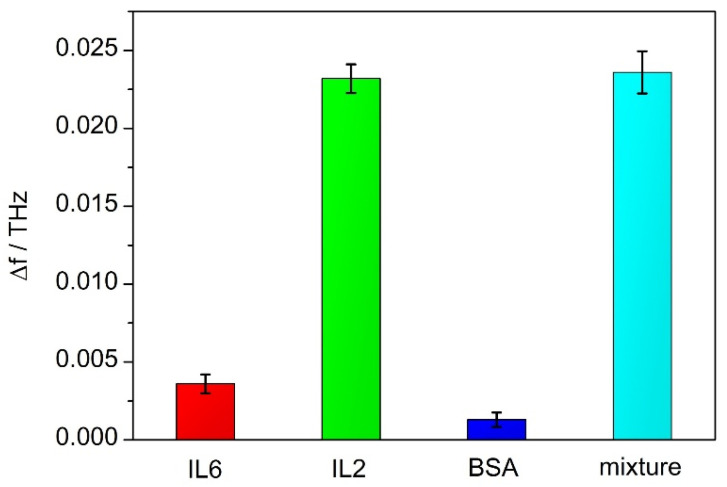
The resonance peak shifts ∆f of the different antigen devices modified with the IL-2 antibody.

In order to investigate the specific recognition ability of the antibody-functionalized THz biosensors, pure BSA and a mixture of IL-2, IL6 (Sangon Biotech) and BSA were selected as distractors. We dropped four solutions on the surfaces of the devices functionalized by the IL-2 antibody, respectively, 1 μg/mL IL6 solution, 1 μg/mL IL-2 solution, 1 μg/mL BSA solution and the mixture solution. Then we dried the surfaces of the devices according to the above method. The resonance peak shifts ∆f of these devices and error bars are shown in Figure 8. The experiments were repeated three times.

The BSA solution and the IL6 solution lead to no resonance peak redshift for the devices modified by the IL-2 antibody. This result demonstrated that the two antibodies of IL-2 were specific for the antigen. 

## 4. Conclusions

In this paper, an all-dielectric metamaterial THz biosensor based on a double-gap ring column array with high sensitivity and a low limit of detection for cytokines has been designed and fabricated successfully. Contributing to the Mie resonance and EIT effect, ultralow loss properties within the THz band is obtained. Since the functional layer comprises single-crystal silicon, the device reliability is improved compared to other polycrystalline silicon-based metamaterial devices, avoiding the use of the EBL exposure process, downgrading costs and facilitating mass production. The device exhibits a low limit of detection of about 100 pg/mL for cytokine IL-2 and shows a Q factor as high as 35 at 0.82 THz resonance. Furthermore, it is experimentally shown that there is an approximate linear relationship between the logarithm of the sample concentration and the resonance peak shift over a wide range of 100 pg/mL to 1 μg/mL. This makes the application of biosensors more convenient for the detection of various antigen proteins in the THz regime. This study has important implications for the detection of cytokines in serum.

## Data Availability

Data are contained within the article.
